# Water Quality and Associated Human Health Risk Assessment Related to Some Ions and Trace Elements in a Series of Rural Roma Communities in Transylvania, Romania

**DOI:** 10.3390/foods13030496

**Published:** 2024-02-04

**Authors:** Călina Creța, Cristina Horga, Mariana Vlad, Vlad-Alexandru Pănescu, Victor Bocoș-Bințințan, Maria-Virginia Coman, Mihaela Cătălina Herghelegiu, Vidar Berg, Jan Ludvig Lyche, Mihail Simion Beldean-Galea

**Affiliations:** 1 Cluj Public Health Regional Centre, National Institute of Public Health, 6 Pasteur Str., RO-400349 Cluj-Napoca, Romania; 2 Faculty of Environmental Science and Engineering, Babeş-Bolyai University, 30 Fântânele Str., RO-400294 Cluj-Napoca, Romania; 3 “Raluca Ripan” Institute for Research in Chemistry, Babeş-Bolyai University, 30 Fântânele Str., RO-400294 Cluj-Napoca, Romania; 4 Faculty of Veterinary Medicine, Norwegian University of Life Sciences, 1433 Ås-Oslo, Norway

**Keywords:** ions, trace elements, drinking water, human health risk, rural Roma communities

## Abstract

This research aims to assess the content of some ions and trace elements in water sources in 24 rural Roma communities in Transylvania in order to assess the human health risk associated with exposure to such elements and ions. To this end, eight ions (F^−^, Cl^−^, Br^−^, NO_2_^−^, NO_3_^−^, SO_4_^2−^, PO_4_^3−^, NH_4_^+^) and ten trace elements (Cr, Ni, As, Pb, Cd, Mn, Cu, Zn, Fe, and Hg) were determined in 71 water samples by ion chromatography coupled with a conductivity detector for ions and atomic absorption spectrophotometry for all trace elements. General parameters were also determined. Non-conformity (as number of samples), according to the EU Drinking Water Directive, was observed as follows: pH (7), EC (7), hardness (1), oxidizability (15), Cl^−^ (4), NO_3_^−^ (30), SO_4_^2−^ (6), Fe (16), Mn (14), As (3), and Ni (1 sample). The incidence of ions was Cl^−^ (71), SO_4_^2−^ (70), F^−^ (67), NO_3_^−^ (65), NH_4_^+^ (21), Br^−^ (10), PO_4_^3−^, and NO_2_^−^ (1 sample) and for trace elements, Mn (59), Fe (50), As (38), Ni (32), Cu (29), Zn (28), Cd (12), Cr (11), and Pb (3 samples). Hg was not detected. Non-carcinogenic (HI) values exceeded one for As in 13 Roma communities, with higher values for children than for adults. For NO_3_^−^, the HI values were >1 in 12 for adults and 14 communities for children. The carcinogenic risk (CR) for As through ingestion ranged from 0.795 to 3.50 × 10^−4^ for adults and from 1.215 to 5.30 × 10^−4^ for children. CR by dermal contact was in the range of ×10^−6^ both for adults and children.

## 1. Introduction

Water is considered a primary need [1], which is both limited [2] and essential for sustaining life [3]; therefore, water quality is crucial for the health and well-being of a country’s inhabitants. Since agriculture, urbanization, industrialization, and other human activities have increased, the general quality of both surface and underground water has suffered [4].

Groundwater contaminants mainly include anions, cations, and toxic metals [2], depending on the geological structures and anthropogenic activities in the region [5]. Some of them, in small amounts, are essential for living organisms and for humans, such as chloride (Cl^−^), potassium (K^+^), sodium (Na^+^), magnesium (Mg^2+^), copper (Cu^2+^), zinc (Zn^2+^), and iron (Fe) [2,5]. 

One of the most important and widespread contaminants in groundwater worldwide is nitrate (NO_3_^−^), which has high solubility, mobility, and stability in water [6,7,8]. Possible sources of nitrate include animal waste, use of nitrogen-containing fertilizers (also a source of nitrites (NO_2_^−^)) [9], and irrigation using wastewater [7,8]. The consumption of water with high concentrations of nitrates and nitrites, especially among infants, leads to methemoglobinemia (“blue baby syndrome”) [8]; among adults, it can cause gastric cancer [9]. In the digestive tract, nitrate is converted to nitrite [10]. Within the European Union, the Drinking Water Directive (EU/2020/2184) [11] sets the standards for nitrate at 50 mg/L and for nitrite at 0.5 mg/L for water intended for human consumption. 

Bromine is an element present in the Earth’s crust and therefore automatically occurs in water. Following the water disinfection process, it can react with organic matter and form brominated disinfection by-products, which are carcinogenic [12]. The threshold for bromate in the drinking water in the European Union is set at 10 μg/L [11]. Although in low concentrations (<0.5 mg/L), fluoride (F^−^) is necessary for bone mineralization and the formation of dental enamel, while in high concentrations (>1.5 mg/L) it can cause dental and skeletal fluorosis [5,8]. For this reason, in the European Union, the concentration of fluoride in drinking water must not exceed the threshold of 1.5 mg/L [11]. 

Nitrate, chloride, and sulfate (SO_4_^2−^) are considered indicators of anthropogenic contamination; sodium and chloride indicate salinization, bicarbonate (HCO_3_^−^) shows acidification or water hardening, while fluoride indicates an intensive evaporation [13]. In the European Union, the concentrations of chloride and sulfate in drinking water must not exceed the threshold of 250 mg/L [11].

Along with anions and cations, toxic metals are usually present in water, endangering the health of both humans and aquatic organisms. Toxic metals are often associated with anthropogenic activities (like agricultural practices, mining and smelting activities, domestic and industrial wastewaters, and even emissions due to traffic) and with natural sources (erosion and weathering of crustal materials) [14,15,16,17,18]. High temperatures and low pH values allow the toxic metals to be more easily released into the water [19]. Because of these reasons, toxic metals are a significant global concern, being ubiquitous, persistent, non-biodegradable, and bioaccumulative [18,20,21]. Long-term exposure to water contaminated with toxic metals can drastically affect the normal functioning of the human body, since they interact with biological molecules containing nitrogen, oxygen, and sulfur, triggering structural changes and functional effects. This can eventually lead to central nervous system disorders, skin lesions and blood vessel damage, immune system dysfunction, gastrointestinal and kidney problems, and sometimes cancer [22]. For example, the inorganic form of arsenic is much more toxic than the organic form [5]; exposure can lead to dermal lesions, cardiovascular disease, as well as skin, bladder, and lung cancer [23]. Cadmium poisoning can produce kidney, lung, and liver dysfunction, bone fracture, and cancer [21]. Lead intake can cause anemia, central nervous system dysfunctions, as well as kidney and fertility problems [1,21,23]. Nickel can cause skin irritation, as well as kidney, liver, and brain dysfunctions, while copper ingestion causes dizziness, nausea, and respiratory and gastrointestinal problems. Mercury intake may lead to the development of neurological injuries, and chromium can generate skin irritation and kidney dysfunction [16,21]. Ingesting zinc can lead to digestive disorders and even death [24]. In the European Union, the thresholds of heavy metals as contaminants present in drinking water are As 10 μg/L; Cd 5 μg/L; Cr 50 μg/L; Cu 2 mg/L; Pb 10 μg/L; Hg 1 μg/L; and Ni 20 μg/L [11]. 

Evaluating health risk by investigating some of the anions and toxic metals in water is essential to determine the probable illnesses caused by drinking water and to protect public health as well; this risk assessment is being carried out all around the world [1,3,7,8,10,15,19]. The most commonly applied methods are chronic daily intake, hazard quotient, and total carcinogenic risk [19,25,26,27,28,29,30,31,32,33,34]. Human health risk assessment is defined by the United States Environmental Protection Agency (US EPA) [25] as a systematic approach to assess the potential health effects of exposure to certain harmful substances that are present in polluted environmental media [25,26]. Simply said, it is a systematic model for quantitative or semi-quantitative description of the negative effects of exposure to harmful substances [8].

In the less developed areas of Romania, including those inhabited by Roma rural communities, wells are fed with underground water and provide drinking water to a large part of that community’s population. Groundwater is used without any prior treatment or analysis, therefore posing a significant health risk of exposure to contaminants. According to the European Union Drinking Water Directive [11], Romania, as a member state, must take all the measures to ensure that the water for human consumption is free of chemicals, parasites, and microorganisms that could endanger human health; also, ensuring access to water must be, as a main sustainable development objective, a priority in the case of marginalized population categories such as the Roma.

The main objective of this study was to evaluate water contamination with certain ions and toxic metals in a series of rural Roma communities in Transylvania, Romania, and then to assess the human health risk due to water consumption. In addition, the findings of this study will help to design and implement a set of guide recommendations for improving rural drinking water quality for the residents of the mentioned communities. 

## 2. Material and Methods

### 2.1. Samples and Sampling Areas

**Figure 1:**
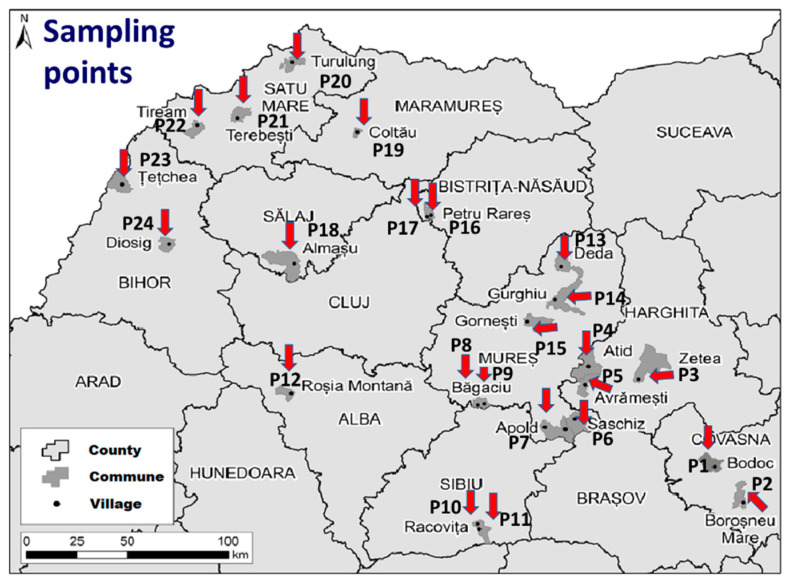
Map indication of all 24 water sampling points in the investigated area.

A total of 71 water samples were collected in 24 rural Roma communities in Transylvania, Romania (Figure 1). 

From each location, water samples were collected from the most used drinking water sources as follows: P1—1 sample; P2—3 samples; P3—2 samples; P4—3 samples; P5—3 samples; P6—4 samples; P7—4 samples; P8—1 sample; P9—3 samples; P10—2 samples; P11—2 samples; P12—4 samples; P13—2 samples; P14—5 samples; P15—3 tests; P16—2 samples; P17—2 samples; P18—3 samples; P19—4 samples; P20—2 samples; P21—4 samples; P22—4 samples; P23—4 samples; P24—4 samples.

The water samples were collected in 1 L brown glass bottles between October and November 2021. After sampling, the samples were transported to the laboratory in refrigerated bags and analyzed within a maximum of 48 h after sampling. Until the analysis, the samples were kept at a temperature of 5 °C. Before analyses, the water samples were filtered through filters with a pore size of 0.45 µm.

### 2.2. Chemicals and Reagents

For the proposed experiments, a certified reference mixture containing 10 mg/L F^−^, 20 mg/L Cl^−^, Br^−^, NO_2_^−^, NO_3_^−^, and 30 mg/L SO_4_^2−^, PO_4_^3−^ and calibration standard (IV) of 23 components (Ag, Al, B, Ba, Bi, Ca, Cd, Co, Cr, Cu, Fe, Ga, In, K, Li, Mg, Mn, Na, Ni, Pb, Sr, Tl, Zn in HNO_3_ 2%) at a concentration of 1000 mg/L each were purchased from CPA Chem (Bogomilovo, Bulgaria). Ammonium (NH_4_^+^) CRM at a concentration of 1000 mg/L was acquired from Inorganic Ventures (Christiansburg, VA, USA). Ultrapure HNO_3_ 65% and HCl 30% and standard for As at a concentration of 1000 mg/L in HNO_3_ 2–3% were purchased from Merck (Darmstadt, Germany) and methanesulfonic acid solution 3 mM, sodium carbonate 4.5 mM, sodium bicarbonate 1.4 mM, and sodium boride 98% were acquired from Thermo Scientific (Milan, Italy). Milli-Q water was prepared using a Milli-Q-Plus ultrapure water system (Millipore, Milford, MA, USA). 

### 2.3. Analysis Methods

After the samples arrived at the laboratory, a series of physicochemical parameters, including pH, electrical conductivity (EC), carbonate and non-carbonate hardness, and oxidizability indices, were measured. pH and EC were measured using a multiparameter (Hanna Instrument, Cluj-Napoca, Romania). Before each series of measurements, the pH electrode was calibrated using two buffer solutions of pH 4 and 9, respectively, while the conductometer was calibrated using standards of 450 μS/cm and 1000 μS/cm. Carbonate and non-carbonate hardness and oxidizability indices were determined by titrimetric methods, using standard solutions of 0.1 N HCl, 0.1 N EDTA, and 0.1 N KMnO_4_, respectively. Total hardness was expressed as the sum of carbonate and non-carbonate hardness and is given in German degrees (1 German degree = 10 mg CaO).

The determination of anions (F^−^, Cl^−^, Br^−^, NO_2_^−^, NO_3_^−^, SO_4_^2−^, PO_4_^3−^) was carried out by ion exchange chromatography using a Shimadzu ion chromatograph equipped with a high-pressure pump, conductometric detector, thermostat for columns, autosampler, and suppressor. The separation was carried out on a Dionex Ion Pac AS22 column (4 × 250 mm), using a mobile phase containing 4.5 mM sodium carbonate, 1.4 mM sodium bicarbonate. The flow rate of the mobile phase was 1 mL/min, and the samples were injected with a Shimadzu SIL-10 ADVP autosampler.

Ammonium was also determined by ion chromatography, using a Dionex Ion Pac SCS1 column (4 × 250 mm), with a mobile phase of 1 mL/min of 3 mM methanesulfonic acid solution, without suppression.

The quantification of the selected ions was carried out based on the calibration curves of five standard solutions of different concentrations prepared by successive dilutions of the standard mixtures. Quantification limits for the studied ions were F^−^—0.09, Cl^−^—0.36, Br^−^—0.39, NO_2_^−^—0.05, NO_3_^−^—0.45, SO_4_^2−^—0.43, PO_4_^3−^—0.58, and NH_4_^+^—0.07 mg/L.

Elements determinations (Cr, Ni, As, Pb, Cd, Mn, Cu, Zn, Fe, Hg) were made by atomic absorption spectrophotometry (AAS) using a Shimadzu AAS model AA-6300 with atomization in a graphite furnace (Cr, Ni, As, Pb, Cd, Mn), flame atomization (Cu, Zn, Fe), and hydride generator (Hg). The selected wavelengths for metals were Cr (λ = 357.9 nm), Ni (λ = 232. nm), As (λ = 193.7 nm), Pb (λ = 283.3 nm), Cd (λ = 228.8 nm), Mn (λ = 279.5 nm), Cu (λ = 357.9 nm), Zn (λ = 213.9 nm), Fe (λ = 248.3 nm), Hg (λ = 228.8 nm). The quantitative determinations were made based on the calibration curves of 4 standard solutions of different concentrations prepared by successive dilutions of the standard mixtures. Quantification limits for the studied elements were Cr—0.78, Ni—1.96, As—1.80, Pb—1.52, Cd—0.1, Mn—0.73, Hg—0.29, Cu—10, Zn—20, and Fe—46 µg/L. The relative standard deviation of the instrumental analyses for three successive determinations was below 7.45%.

For the determination of anions and ammonium, the water samples were filtered through filters with a pore size of 0.45 µm, and for the determination of metals, the water samples were acidified to pH < 2 with 65% nitric acid.

### 2.4. Health Risk Assessment

Human health risk assessment involves estimating and quantifying the probability of occurrence of an event and the magnitude of its effect on health with a given exposure over a defined period. To this end, the United States Environmental Protection Agency (US EPA) has proposed a human health risk assessment model [25] widely used worldwide [26,27,28,29,30,31] which is based on four steps as follows: hazard identification, exposure assessment, response to a certain dose, and risk characterization [26].

In this study, the health risk was assessed by non-carcinogenic and carcinogenic risk of pollutants according to the US EPA recommendation [25].

The ingestion route and dermal contact were considered for estimating the exposure of the local population. The average daily doses from ingestion (ADD_ing_) and dermal absorption (ADD_dermal_) of a pollutant via water as the exposure pathways are expressed in (μg/kg/day) and were estimated by the following equations [26,28,29,31,33]:

(1) $${ADD}_{ing}=\frac{C_{w}\times IR\times EF\times ED}{BW\times AT}$$

(2) $${ADD}_{dermal}=\frac{C_{w}\times SA\times K_{p}\times ET\times EF\times ED\times 10^{-3}}{BW\times AT}$$

where ADD_ing_ and ADD_dermal_ represent the average daily doses via ingestion and dermal adsorption (μg/kg/day); BW is the average body weight (kg); AT is the average time of exposure (days); C_w_ is the average concentration of heavy metals in water (μg/L); IR is the ingestion rate (L/day); EF is the exposure frequency (days/year); ED is the exposure duration (year); SA is the exposed skin area (cm^2^); ET is the exposure time (h/day); and K_p_ is the dermal permeability coefficient in water (cm/h). All exposure parameters were referenced from EPA and the scientific literature [25,27,28].

The potential non-carcinogenic risk via ingestion and dermal routes was assessed by hazard quotient (HQ) and hazard index (HI) using the following equations [28,33,34]:

(3) $${HQ}_{ing}=\frac{{ADD}_{ing}}{R_{f}D_{oral}}$$

(4) $${HQ}_{dermal}=\frac{{ADD}_{dermal}}{R_{f}D_{dermal}}$$

(5) $$HI=\sum ({HQ}_{ing}+{HQ}_{dermal})$$

where R_f_D_oral_ and R_f_D_dermal_ are the oral and dermal toxicity reference doses for a specific metal. 

The carcinogenic risk (CR) was estimated only for As and was calculated using the following equations [28,33]:

(6) $${CR}_{ing}={ADD}_{ing}\times {SF}_{ing}$$

(7) $${CR}_{dermal}={ADD}_{dermal}\times {SF}_{dermal}$$

where SF represents the carcinogenic slope factors via ingestion and dermal exposure. 

The values of variables considered to estimate ADD, HQ, HI, and CR are given in Table 1 [25,26,27,28,29,30,31,32,33].

## 3. Results and Discussion 

### 3.1. General Characteristics of Water Quality

Minimum, maximum, and average values of physicochemical parameters, anions, ammonium, and trace element concentration of analyzed water samples as a mean for each selected rural Roma community, as well as the maximum allowable concentration (MAC) established by the EU Drinking Water Directive [11], are given in Table 2. NO_2_^−^ and PO_4_^3−^ were found in one sample at a concentration of 5.77 and 4.57 mg/L and do not exceed MAC.

From the 71 water samples analyzed, non-conformities with the limits established by the EU Directive were detected as follows: pH—7, EC—7, hardness—1, oxidizability—15, Cl^−^—4, NO_3_^−^—30, SO_4_^2−^—6, Ni—1, As—3, Mn—14, and Fe—16 samples.

For a better visualization of the amplitude of the concentrations found in each selected Roma community, the ions and metals are further treated separately.

### 3.2. Anions and Ammonium Content in Water Samples

The highest incidence of anions in the samples analyzed was found for chloride (71 samples), followed by sulfate (70 samples), fluoride (67 samples), nitrate (65 samples), bromide (10 samples), and phosphate and nitrite (1 sample). Ammonium was detected in 21 analyzed water samples (Supplementary Material Table S1). In order to estimate the population’s exposure to anions and ammonium through water consumption, the mean value obtained for each locality was taken into account (Table 3).

From Table 3, it can be concluded that Cl^−^ exceeds the MAC in two communities, NO_3_^−^ in fourteen communities, and SO_4_^2−^ in three communities. F^−^ and NH_4_^+^ do not exceed MAC in any selected community. Considering that the nitrite anion NO_2_^−^ was found in only one sample, we can assume that the nitrate (NO_3_^−^) has a geological origin. 

### 3.3. Trace Element Content in Water Samples

The highest incidence of trace elements in the analyzed samples was found for Mn (59 samples), followed by Fe (50 samples), As (38 samples), Ni (32 samples), Cu (29 samples), Zn (28 samples), Cd (12 samples), Cr (11 samples), and Pb (3 samples) (Table S1). Hg was not detected in any analyzed sample. For a better estimate of the population’s exposure to metals through water consumption, the mean value obtained for each locality was considered (Table 4).

The results of the analyses carried out show that for Cr, Ni, As, Pb, Cd, and Cu, there are no MAC exceedances in any selected community. There are exceedances of the MAC for Mn in six communities and for Fe in seven communities. No MAC is established for Zn. Considering that in those investigated areas there were/are no anthropic activities that may generate heavy metals, we can assume that all investigated trace elements had a geological source.

Since the water sources are used in the studied communities both as a source of drinking water and as sources of domestic water, a simple comparison of the values found with the MAC is not sufficient. In order to study the effect on human health due to a long-term exposure to the studied elements, an evaluation of non-carcinogenic and carcinogenic risk is necessary.

### 3.4. Health Risk Assessment

#### 3.4.1. Non-Carcinogenic Risk Assessment

According to the model recommended by the US EPA [25], the non-carcinogenic risk of trace elements and NO_3_^−^ in water through ingestion and dermal routes was calculated and used as a health risk assessment tool (Tables S2–S4). The minimum, maximum, and average results of the HQ_ing_, HQ_dermal_, and HI results calculated using the average values of the elements measured in each Roma community are summarized in Table 5. 

Apart from As and NO_3_^−^, the HI values were within the safety limits (<1), which indicates that the water from investigated rural Roma communities does not present a health risk [26] from the point of view of the investigated metals (Supplementary Materials Table S4). For As, in 13 of the 24 investigated Roma communities, the HI value is >1, which means that a significant health hazard occurs in these communities (Table S4) [26]. The risk is higher for children than for adults, with the calculated HI values being higher in all 13 cases. In the case of NO_3_^−^, the HI values for adults are >1 in 12 Roma communities, compared to 14 of the 24 Roma communities in the case of children.

HQ values of metals and NO_3_^−^ through dermal contact for adults and children were below 1 × 10^−3^ (adults: 0.02 to 98.45 × 10^−3^; children: 0.01 to 57.83 × 10^−3^) (Table S3), indicating that the investigated elements presented a low hazard through dermal absorption [25,26].

Except for As and NO_3_^−^, the HQ values of the investigated metals through the ingestion route ranged from 0.002 to 0.774 for adults and 0.002 to 1.129 (for Mn) (Table S2), indicating that ingestion exposure has a potential non-carcinogenic risk [26], being the main contributor to HI. For As, HQ_ing_ values range from 1.76 to 7.76 for adults and from 2.70 to 11.76 for children. For NO_3_^−^, HQ_ing_ values range from 0.003 to 6.13 for adults and from 0.004 to 9.32 for children. Values exceeding 1 indicate that the water sources present a significant non-carcinogenic risk for consumers [25,26].

#### 3.4.2. Carcinogenic Risk Assessment

Among the elements studied, only As presents a carcinogenic risk, which is why the CR was calculated only for this element. CR was calculated for both ingestion and dermal contact routes (Table S5).

The obtained results show that in the 13 Roma communities in which As was identified, CR by ingestion for adults varied between 0.795 and 3.50 × 10^−4^ and, for children, 1.215 and 5.30 × 10^−4^ (Table 6). CR by dermal contact ranged from 1.01 to 4.43 × 10^−6^ for adults and from 0.61 to 2.59 × 10^−6^ for children (Table 6).

While the CR_dermal_ values were within the EPA 2004 recommended safety limits (<1 × 10^−6^), the CR_ing_ values were 10^2^ higher (1 × 10^−4^) but still within the acceptable CR range recommended by the US EPA (10^−6^ to 10^−4^) [25]. However, taking into consideration its carcinogenic risk, As can represent a risk for populations exposed through drinking water, which requires, in the future, a permanent monitoring of water sources used by rural Roma communities.

## 4. Conclusions

Twenty-four sampling points, belonging to a variety of rural Roma communities located in 10 counties in Transylvania (the north-western part of Romania), were investigated for (a) a series of five anions and one cation, of which the most relevant was the nitrate (NO_3_^−^), and (b) a series of nine trace elements (Cr; Ni; As; Pb; Cd; Mn; Cu, Zn; and Fe). 

Our investigations revealed that for Cr, Ni, As, Pb, Cd, and Cu, there were no MAC exceedances in any selected Roma community; however, exceedances of the MAC were found for manganese (Mn) and for iron (Fe), in six and in seven communities, respectively. 

Except for As and NO_3_^−^, we found that hazard index (HI) values were well within the safety limits (HI < 1), which suggests that water from investigated rural Roma communities does not present a health risk associated with the investigated chemical species. On the other hand, for As the HI was >1 in 13 of the 24 investigated Roma communities, which means that a significant health hazard occurs in these communities; also, the risk is even higher for children than for adults, since the calculated HI is higher in all 13 communities. In the case of nitrate (NO_3_^−^), the HI values for adults have values > 1 in 12 Roma communities, but HI values for children were >1 in 14 of the 24 studied Roma communities. 

Hazard quotient (HQ) values for both metals and nitrate (NO_3_) through dermal contact were <1 × 10^−3^ (adults: 0.02 to 98.45 × 10^−3^; children: 0.01 to 57.83 × 10^−3^), which indicates that these chemical species present a low hazard through dermal absorption. 

Hazard quotient (HQ) through ingestion for the investigated metals suggests a relevant potential non-carcinogenic risk and is a main part of the hazard index (HI). However, the HQ_ing_ values > 1 found for As (from 1.76 to 7.76 for adults, and from 2.70 to 11.76 for children) and for nitrate (NO_3_^−^) (from 0.003 to 6.13 for adults, and from 0.004 to 9.32 for children) indicate a significant non-carcinogenic risk for water consumers.

Carcinogenic risk (CR) is known currently only for As, and it was calculated for both dermal contact and ingestion. The determined ingestion CR values for the 13 Roma communities for which As was found in water were from 0.795 to 3.50 × 10^−4^ (for adults) and from 1.215 to 5.30 × 10^−4^ (for children), while the dermal contact CR values were from 1.01 to 4.43 × 10^−6^ (for adults) and from 0.61 to 2.59 × 10^−6^ (for children). All these CR values are below the recommended US EPA thresholds, but one must take into consideration that, due to the known carcinogenic potential of As, a permanent, continuous monitoring of those water sources must be performed.

## Figures and Tables

**Table 1:** The values of the variables used for estimation of ADD, HI, and CR.

Parameters	Abbreviation	Units	Values	References
Body weight	BW	kg	70 (adult); 15 (child)	[28]
Average time	AT	days (d)	ED × 365 (non-carcinogenic)	[25,27,28]
Exposure duration	ED	year (y)	30 (adult); 6 (child)	[27,28]
Exposure frequency	EF	d/y	350	[25,27,28]
Exposure time	ET	h/d	0.58 (adult), 1 (child)	[28]
Ingestion rate	IR	L/d	2 (adult); 0.64 (child)	[28]
Exposed skin area	SA	cm^2^	18,000 (adult), 6600 (child)	[28]
Dermal permeability coefficient	K_p_	cm/h	1 × 10^−3^ (As, Cd, Cu, Mn, Fe); 2 × 10^−3^ (Cr); 1 × 10^−4^ (Pb); 2 × 10^−4^ (Ni); 6 × 10^−4^ (Zn), 1 × 10^−3^ (NO_3_^−^)	[25,27,28]
Ingestion reference dose	RfD_ing_	µg/kg/d	0.03 (As), 0.5 (Cd), 3.0 (Cr), 40 (Cu), 24 (Mn), 20 (Ni), 1.4 (Pb), 300 (Zn), 300 (Fe), 1600 (NO_3_^−^)	[25,27,28]
Dermal reference dose	RfD_dermal_	µg/kg/d	0.285 (As); 0.025 (Cd); 0.075 (Cr); 12 (Cu); 0.96 (Mn); 0.8 (Ni); 0.42 (Pb); 60 (Zn); 45 (Fe), 800 (NO_3_^−^)	[25,27,28]
Carcinogenic slope factor via ingestion	SF_ing_	µg/kg/d	0.0015 (As)	[25,28]
Dermal contact slope factor	SF_dermal_	µg/kg/d	0.0036 (As)	[25,28]

**Table 2:** Values of physicochemical parameters, anions, ammonium, and trace element concentration of water samples in the study area and drinking water standard specifications given by EU Directive [11].

Parameter	Units	Minimum	Maximum	Average	Exceedance	MAC *
pH	-	5.49	8.59	6.87	7	6.5–9.5
EC	μS/cm	100	140,000	4079	7	2500
Hardness	degree	3.64	64.7	25.5	1	>5
Oxidizability	mg/L O_2_	0.32	21.17	4.63	15	5
F^−^	mg/L	0.03	0.99	0.15	0	1.5
Cl^−^	mg/L	0.41	497.98	80.18	4	250
Br^−^	mg/L	0.02	0.28	0.15	-	-
NO_3_^−^	mg/L	0.17	612.45	81.23	30	50
SO_4_^2−^	mg/L	4.24	963.54	107.69	6	250
NH_4_^+^	mg/L	0.05	0.8	0.17	0	0.5
Cr	µg/L	0.78	8.24	2.15	0	25
Ni	µg/L	2.00	20.39	4.48	1	20
As	µg/L	1.81	14.02	4.85	3	10
Pb	µg/L	1.61	1.99	1.76	0	5
Cd	µg/L	0.11	0.53	0.2	0	5
Mn	µg/L	0.83	792.8	136.65	14	50
Cu	µg/L	0.011	0.18	0.03	0	2000
Zn	µg/L	21.2	239.9	66.56	-	-
Fe	µg/L	7.14	1111.1	222.79	16	200

“*” values taken from [11].

**Table 3:** Mean values of the ions in the analyzed water samples for each selected community.

Sampling Point	Concentration (mg/L)					
	F^−^	Cl^−^	Br^−^	NO_3_^−^	SO_4_^2−^	NH_4_^+^
P1	-	497.9	-	62.38	64.87	-
P2	0.23	101.3	-	94.84	64.59	0.05
P3	0.07	1.31	0.02	1.15	15.43	-
P4	0.14	71.74	0.07	73.03	82.82	-
P5	0.12	66.85	0.03	20.66	61.47	-
P6	0.08	22.93	-	15.57	108.6	0.10
P7	0.06	39.57	-	52.00	56.86	0.00
P8	0.27	12.53	-	71.10	693.1	0.11
P9	0.15	45.40	-	31.84	81.69	0.09
P10	0.13	21.58	-	51.84	41.22	-
P11	0.16	19.62	-	8.48	77.21	-
P12	0.06	2.48	-	4.63	11.46	0.11
P13	0.07	12.07	-	26.90	23.91	0.175
P14	0.13	112.1	-	91.41	104.5	0.10
P15	0.40	80.48	0.23	34.15	68.25	0.08
P16	0.16	304.1	0.28	189.7	285.4	-
P17	0.14	133.1	-	268.0	142.08	-
P18	0.24	52.44	-	105.9	416.2	-
P19	0.15	112.5	-	71.98	94.15	-
P20	0.11	230.7	0.18	152.4	111.7	-
P21	0.17	2.97	-	0.17	6.04	0.10
P22	0.17	60.87	0.20	66.30	107.5	0.45
P23	0.12	48.87	0.24	24.72	62.38	0.25
P24	0.25	190.4	-	363.5	214.6	0.47
MAC * (mg/L)	1.50	250.00	-	50.00	250.0	0.50

“*” values taken from [11], “-” not detected.

**Table 4:** Mean values of the metals in the analyzed water samples for each selected community.

Sampling Point	Concentration (µg/L)								
	Cr	Ni	As	Pb	Cd	Mn	Cu	Zn	Fe
P1	-	5.22	-	-	-	-	-	154.50	107.9
P2	2.09	20.39	-	1.99	-	528.6	-	53.55	54.3
P3	-	-	-	-	-	2.66	-	45.20	104.0
P4	-	3.07	-	-	-	47.26	15.00	-	87.2
P5	-	2.42	1.97	-	-	14.26	25.00	40.10	95.8
P6	-	4.32	3.57	-	-	276.9	73.67	128.83	615.5
P7	-	2.71	4.83	-	0.18	16.40	21.50	36.30	-
P8	2.13	2.79	-	-	-	49.8	-	-	137.5
P9	1.09	-	-	-	-	6.46	-	25.20	112.9
P10	-	2.09	-	-	-	12.15	-	32.80	342.7
P11	-	-	-	-	-	7.84	-	-	357.5
P12	1.91	3.28	-	-	-	18.40	12.00	-	452.5
P13	-	-	2.68	-	-	12.14	-	23.50	67.3
P14	-	-	3.19	-	0.11	10.51	-	-	100.0
P15	-	2.62	-	-	-	7.80	17.00	44.20	197.1
P16	-	5.40	3.10	-	0.39	-	22.50	-	-
P17	-	2.83	3.18	-	0.11	-	20.50	33.80	50.0
P18	1.43	5.50	-	-	0.17	9.92	26.67	40.53	121.1
P19	2.13	3.07	2.94	1.61	-	5.25	24.50	92.15	122.4
P20	0.79	11.12	3.85	-	-	68.25	25.00	79.10	327.9
P21	-	-	7.23	-	-	660.9	-	-	227.1
P22	-	2.25	5.98	-	-	526.9	11.50	28.10	499.6
P23	8.24	-	5.12	-	-	9.95	20.00	-	-
P24	1.54	6.64	8.62	1.67	0.17	192.0	14.50	90.63	185.9
MAC * (µg/L)	25	20	10	5	5	50	2000	-	200

“*” values taken from [11], “-” not detected.

**Table 5:** The minimum, maximum, and average results of the HQ_ing_, HQ_dermal_, and HI of the investigated metals and NO_3_^−^ in analyzed water samples.

Elements	Person	HQ_ing_			HQ_dermal_ × 10^−3^			HI		
		Min	Max	Average	Min	Max	Average	Min	Max	Average
Cr	Adult	0.007	0.074	0.021	3.07	31.47	9.07	0.01	0.105	0.03
	Child	0.003	0.113	0.032	1.73	18.4	5.3	0.012	0.131	0.038
Ni	Adult	0.003	0.028	0.007	0.08	0.73	0.18	0.003	0.028	0.007
	Child	0.004	0.042	0.01	0.05	0.43	0.11	0.004	0.042	0.01
As	Adult	1.76	7.76	3.89	0.98	4.32	2.17	1.77	7.77	3.89
	Child	2.70	11.76	5.91	0.6	2.53	1.28	2.70	11.76	5.91
Pb	Adult	0.031	0.039	0.034	0.05	0.07	0.06	0.031	0.039	0.034
	Child	0.006	0.059	0.051	0.02	0.05	0.03	0.047	0.059	0.051
Cd	Adult	0.006	0.022	0.011	0.8	2.4	1.13	0.007	0.024	0.012
	Child	0.003	0.032	0.016	0.4	1.2	0.6	0.01	0.033	0.016
Mn	Adult	0.003	0.744	0.133	0.4	98.45	17.62	0.003	0.842	0.151
	Child	0.005	1.129	0.202	0.23	57.83	10.35	0.005	1.187	0.212
Cu	Adult	0.008	0.05	0.016	0.14	0.88	0.28	0.008	0.051	0.016
	Child	0.002	0.126	0.04	0.08	0.52	0.16	0.02	0.126	0.04
Zn	Adult	0.002	0.014	0.005	0.03	0.22	0.09	0.002	0.014	0.005
	Child	0.003	0.021	0.008	0.02	0.13	0.05	0.003	0.021	0.008
Fe	Adult	0.005	0.055	0.019	0.16	1.96	0.66	0.005	0.057	0.019
	Child	0.007	0.084	0.028	0.09	1.15	0.39	0.007	0.085	0.029
NO_3_^−^	Adult	0.003	6.13	1.32	0.02	64.98	14.02	0.003	6.20	1.34
	Child	0.004	9.32	2.01	0.01	38.17	8.24	0.004	9.36	2.02

**Table 6:** The minimum, maximum, and average CR values for As via ingestion and dermal exposure routes.

Person	CR_ing_ for As × 10^−4^			CR_dermal_ for As × 10^−6^		
	Min	Max	Average	Min	Max	Average
Adult	0.795	3.50	1.75	1.0	4.4	2.4
Child	1.215	5.30	2.66	0.6	2.6	1.4

## Data Availability

Data are contained within the article.

## Supplementary Materials

The following supporting information can be downloaded at: https://www.mdpi.com/article/10.3390/foods13030496/s1, Table S1. The concentration on ions (mg/L) and trace elements (µg/L) in the analyzed water samples from selected rural Roma communities; Table S2. The hazard quotient via ingestion route (HQ_ing_) for adults and children of trace elements and nitrate in selected rural Roma communities; Table S3. The hazard quotient via dermal route (HQ_dermal_) expressed as value × 10^−3^ for adults and children of trace elements and nitrate in selected rural Roma communities; Table S4. The hazard index (HI) for adults and children of trace elements and nitrate in selected rural Roma communities; Table S5. The cancer risk (CR) for adults and children of As via ingestion and dermal routes in selected rural Roma communities.
