# Supplementary material for: Water Quality and Associated Human Health Risk Assessment Related to Some Ions and Trace Elements in a Series of Rural Roma Communities in Transylvania, Romania
Source: Foods. 2024 Feb 4;13(3):496. doi: 10.3390/foods13030496 (PMC10855629; doi:10.3390/foods13030496)
Supplement: Supplementary file 1 [file foods-13-00496-s001.zip › foods-2850491-supplementary.pdf]

SUPPLEMENTARY INFORMATION

Water Quality and Associated Human Health Risk Assessment Related to Some Ions and Trace Elements in a Series of Rural Roma Communities in Transylvania, Romania

Călina Creța [1], Cristina Horga [1], Mariana Vlad [1], Vlad-Alexandru Pănescu [2,3], Victor Bocoș-Bințințan [2], Maria-Virginia Coman [3,*], Mihaela Cătălina Herghelegiu [2,3], Vidar Berg [4], Jan Ludvig Lyche [4] and Mihail Simion Beldean-Galea [2,*]

[1] Cluj Public Health Regional Centre, National Institute of Public Health, 6 Pasteur Str., RO-400349 Cluj-Napoca, Romania
[2] Faculty of Environmental Science and Engineering, Babeş-Bolyai University, 30 Fântânele Str., RO-400294 Cluj-Napoca, Romania
[3] "Raluca Ripan" Institute for Research in Chemistry, Babeş-Bolyai University, 30 Fântânele Str., RO-400294 Cluj-Napoca, Romania
[4] Faculty of Veterinary Medicine, Norwegian University of Life Sciences, 1433 Ås-Oslo, Norway
* Correspondence: virginia.coman@ubbcluj.ro (M.-V.C.); simion.beldean@ubbcluj.ro (M.S.B.-G.)

The supplementary information contains 7 pages and includes 5 tables.

List of contents of the supplementary information:

Table S1. The concentration on ions (mg/L) and trace elements (µg/L) in the analysed water samples from selected rural Roma communities
Table S2. The hazard quotient *via* ingestion route (HQ_{ing}) for adults and children of trace elements and nitrate in selected rural Roma communities.
Table S3. The hazard quotient *via* dermal route (HQ_{dermal}) expressed as value x 10^{-3} for adults and children of trace elements and nitrate in selected rural Roma communities.
Table S4. The hazard index (HI) for adults and children of trace elements and nitrate in selected rural Roma communities
Table S5. The cancer risk (CR) for adults and children of As *via* ingestion and dermal routes in selected rural Roma communities

Table S1. The concentration on ions (mg/L) and trace elements (µg/L) in the analysed water samples from selected rural Roma communities

Sample No	F^-	Br^-	Cl^-	NO^{3-}	SO_4^{2-}	NH^{4+}	Cr	Ni	As	Pb	Cd	Mn	Cu	Zn	Fe
P1 – 1	-	-	497.9	62.38	64.87	-	-	5.22	-	-	-	-	-	154.5	107.9
P2 – 1	0.13	-	181.7	63.62	98.04	0.052	-	20.39	-	-	-	612.1	-	31.9	59.6
P2 – 2	0.23	-	35.54	9.13	58.54	-	-	-	-	-	-	445.2	-	-	49.1
P2 – 3	0.32	-	86.65	211.8	37.2	-	2.09	-	-	1.99	-	-	-	75.2	54.3
P3 – 1	0.054	-	1.44	2.04	6.57	-	-	-	-	-	-	2.76	-	45.2	125.2
P3 – 2	0.094	0.022	1.18	0.26	24.29	-	-	-	-	-	-	2.56	-	-	82.8
P4 – 1	0.17	0.11	165.6	193.2	143.3	-	-	3.07	-	-	-	12.89	0	-	76.6
P4 – 2	0.164	0.02	27.53	16.95	55.92	-	-	-	-	-	-	81.63	0	-	97.8
P4 – 3	0.08	-	22.08	8.95	49.22	-	-	-	-	-	-	-	15	-	-
P5 – 1	-	-	54.09	5.63	12.67	-	-	2.42	1.97	-	-	0.91	0	-	104.9
P5 – 2	0.15	0.03	58.73	45.07	82.98	-	-	-	-	-	-	-	0	52.6	98.5
P5 – 3	0.08	-	87.74	11.29	88.75	-	-	-	-	-	-	27.6	25	27.6	83.9
P6 – 1	0.07	-	22.63	0.49	118.6	-	-	3.9	4.05	-	-	452.7	19	52.6	728.1
P6 – 2	0.08	-	17.33	0.52	127	0.11	-	-	-	-	-	-	22	69.2	-
P6 – 3	0.09	-	32.53	40.99	102.7	-	-	4.73	3.09	-	-	21.7	180	153.6	-
P6 – 4	0.07	-	19.23	20.28	86.05	0.08	-	-	-	-	-	356.4	-	239.9	502.8
P7 – 1	-	-	24.29	30.46	39.14	-	-	2.79	8.04	-	0.28	-	18	-	-
P7 – 2	0.09	-	0.64	-	22.71	-	-	2.63	3.97	-	0.11	-	22	21.2	-
P7 – 3	0.03	-	56.38	70.48	68.64	-	-	2.87	3.52	-	0.14		26	51.4	-
P7 – 4	0.06	-	76.95	55.05	96.93	-	-	2.54	3.78	-	-	16.4	20	-	-
P8 – 1	0.27	-	12.53	71.1	693.1	0.11	2.13	2.79	-	-	-	49.8	-	-	137.5
P9 – 1	0.17	-	28.69	29.29	99.89	0.09	-	-	-	-	-	13.65	-	25.2	132.2
P9 – 2	0.16	-	4.63	8.63	35.29	-	-	-	-	-	-	2.86	-	-	100.7
P9 – 3	0.12	-	102.9	57.6	109.9	-	1.09	-	-	-	-	2.86	-	-	105.9
P10 – 1	0.12	-	13.09	32.64	36.2	-	-	2.09	-	-	-	7.21	-	-	312.8
P10 – 2	0.14	-	30.08	71.04	46.24	-	-	-	-	-	-	17.09	-	32.8	372.7
P11 – 1	0.15	-	2.53	0.83	76.53	-	-	-	-	-	-	9.28	-	-	180.4
P11 – 2	0.16	-	36.7	16.12	77.89	-	-	-	-	-	-	6.39	-	-	534.6
P12 – 1	0.046	-	0.47	1.68	4.83	0.15	-	2.42	-	-	-	5.73	13	-	125.3
P12 – 2	0.04	-	0.41	0.46	4.24	0.09	1.91	5.04	-	-	-	36.94	11	-	1111
P12 – 3	0.07	-	7.37	13.1	28.66	0.08	-	2.88	-	-	-	3.46	-	-	7.14
P12 – 4	0.1	-	1.68	3.27	8.09	-	-	2.76	-	-	-	27.47	-	-	566.3
P13 – 1	0.09	-	19.43	36.52	32.28	0.18	-	-	2.81	-	-	4.92	-	-	67.3
P13 – 2	0.05	-	4.7	17.29	15.54	0.17	-	-	2.55	-	-	19.36	-	23.5	-
P14 – 1	0.11	-	13.76	3.13	36.46	0.09	-	-	2.12	-	0.11	3.4	-	-	100
P14 – 2	0.11	-	85.03	51.41	103.9	0.09	-	-	4.8	-	-	34.87	-	-	-
P14 – 3	0.13	-	89.37	108.1	50.93	0.11	-	-	4.2	-	-	6.42	-	-	-
P14 – 4	0.21	-	222.3	169.4	256.4	-	-	-	2.64	-	-	3.65	-	-	-
P14 – 5	0.09	-	149.9	125	74.91	-	-	-	2.2	-	-	4.23	-	-	-
P15 – 1	0.16	0.25	81.2	31.02	74.83	-	-	2	-	-	-	0.83	-	-	138.5
P15 – 2	0.99	0.2	90.47	50.85	91.36	0.08	-	3.23	-	-	-	4.47	-	21.6	213.6
P15 – 3	0.05	-	69.78	20.59	38.56	-	-	2.62	-	-	-	18.1	17	66.8	239.2
P16 – 1	0.17	-	177.5	268.1	150.9	-	-	5.59	1.81	-	0.24	-	23	-	-
P16 – 2	0.15	0.28	430.7	111.4	419.8	-	-	5.21	4.38	-	0.53	-	22	-	-
P17 – 1	0.13	-	191.1	426.9	172.7	-	-	3.11	4.24	-	0.11	-	21	33.8	-
P17 – 2	0.15	-	75.05	109.1	111.4	-	-	2.55	2.12	-	-	-	20	-	50
P18 – 1	0.08	-	17.97	71.26	186.6	-	1.43	-	3.81	-	-	17.09	18	43.7	121.5
P18 – 2	0.55	-	3.34	7.48	98.46	-	-	3.57	1.96	-	-	8.31	15	30.1	142.2
P18 – 3	0.08	-	136	239.2	963.5	-	-	7.42	-	-	0.17	4.35	47	47.8	99.6
P19 – 1	0.097	-	16.2	32.26	22.5	-	-	-	-	-	-	7.41	11	-	49
P19 – 2	0.05	-	85.2	49.26	248.2	-	2.13	-	-	1.61	-	7.48	50	117.9	211.2
P19 – 3	0.215	-	163.6	154.7	71.98	-	-	3.19	2.94	-	-	3.57	17	66.4	133
P19 – 4	0.253	-	185.2	51.62	33.92	-	-	2.95	-	-	-	2.55	20	-	96.2
P20 – 1	-	-	203.1	137.3	82.53	-	0.79	11.12	3.9	-	-	11.2	0	-	68
P20 – 2	0.11	0.18	258.4	167.5	140.8	-	0.78	-	3.79	-	-	125.3	25	79.1	587.7
P21 – 1	0.2	-	3.12	0.17	6.19	0.13	-	-	11.82	-	-	792.8	-	-	194.6
P21 – 2	0.2	-	2.96	-	6.12	0.07	-	-	4.05	-	-	676.4	-	-	525.1
P21 – 3	0.13	-	3.11	-	5.95	-	-	-	7.82	-	-	623.2	-	-	103.8
P21 – 4	0.14	-	2.69	-	5.89	-	-	-	5.23	-	-	551.4	-	-	84.8
P22 – 1	0.12	-	3.16	-	6.24	0.45	-	-	8.11	-	-	330.6	-	-	699.2
P22 – 2	0.25	0.2	115.9	61.03	158.2	-	-	-	2.67	-	-	602.8	-	-	199.2
P22 – 3	0.08	-	3.25	-	-	-	-	-	7.17	-	-	410.9	11	28.1	600.3
P22 – 4	0.22	-	121.1	71.57	158.1	-	-	2.25	-	-	-	763.3	12	-	-
P23 – 1	0.11	-	85	52	104.5	0.11	-	-	4.92	-	-	6.75	-	-	-
P23 – 2	0.15	-	29.36	15.06	46.78	0.38	-	-	6.01	-	-	2.71	-	-	-
P23 – 3	0.09	-	45.79	14.82	51.81	-	8.24	-	4.32	-	-	26.7	20	-	-
P23 – 4	0.13	0.24	35	17.32	46.34	-	-	-	5.21	-	-	3.62	-	-	-
P24 – 1	0.59	-	274	324	261.4	0.14	1.49	4.92	10.29	1.67	0.18	33.69	-	-	-
P24 – 2	0.21	-	184.5	612.4	252.1	0.8	1.59	-	14.02	-	0.19	461.8	-	142.3	465.6
P24 – 3	0.09	-	148.1	156.7	123.7	-	-	6.44	5.38	-	0.18	24.1	16	72.3	46
P24 – 4	0.1	-	154.4	361.3	221.3	-	-	8.55	4.79	-	0.14	248.5	13	57.3	46

“–“ not detected

Table S2. The hazard quotient *via* ingestion route (HQ_{ing}) for adults and children of trace elements and nitrate in selected rural Roma communities.

HQ	Person	P1	P2	P3	P4	P5	P6	P7	P8	P9	P10	P11	P12	P13	P14	P15	P16	P17	P18	P19	P20	P21	P22	P23	P24
Cr	Adult	-	0.019	-	-	-	-	-	0.019	0.010	-	-	0.017	-	-	-	-	-	0.013	0.019	0.007	-	-	0.074	0.014
	Child	-	0.029	-	-	-	-	-	0.029	0.015	-	-	0.026	-	-	-	-	-	0.020	0.029	0.011	-		0.113	0.021
Ni	Adult	0.007	0.028	-	0.004	0.003	-	-	0.004	-	0.003	-	0.004	-	-	0.004	0.007	0.004	0.007	0.004	0.015	-	0.003	-	0.009
	Child	0.011	0.042	-	0.006	0.005	-	0.006	0.006	-	0.004	-	0.007	-	-	0.005	0.011	0.006	0.011	0.006	0.023	-	0.005	-	0.014
As	Adult	-	-	-	-	1.767	3.200	4.333	-	-	-	-	-	2.400	2.867	-	2.800	2.867	-	2.633	3.467	6.500	5.367	4.600	7.767
	Child	-	-	-	-	2.700	4.867	6.600	-	-	-	-	-	3.667	4.367	-	4.233	4.333	-	4.033	5.267	9.867	8.167	7.000	11.767
Pb	Adult	-	0.039	-	-	-	-	-	-	-	-	-	-	-	-	-	-	-	-	0.031	-	-	-	-	0.032
	Child	-	0.059	-	-	-	-	-	-	-	-	-	-	-	-	-	-	-	-	0.047	-	-	-	-	0.049
Cd	Adult	-	-	-	-	-	-	0.010	-	-	-	-	-	-	0.006	-	0.022	0.006	0.010	-	-	-	-	-	0.010
	Child	-	-	-	-	-	-	0.014	-	-	-	-	-	-	0.010	-	0.032	0.010	0.014	-	-	-	-	-	0.014
Mn	Adult	-	0.595	0.003	0.053	0.016	0.312	0.018	0.056	0.007	0.014	0.009	0.021	0.014	0.012	0.009	-	-	0.011	0.006	0.077	0.744	0.593	0.011	0.216
	Child	-	0.903	0.005	0.081	0.024	0.473	0.028	0.085	0.011	0.021	0.013	0.031	0.021	0.018	0.013	-	-	0.017	0.009	0.117	1.129	0.900	0.017	0.328
Cu	Adult	-	-	-	0.010	0.017	0.050	0.015	-	-	-	-	0.008	-	-	0.011	0.015	0.014	0.018	0.017	0.017	-	0.008	0.014	0.010
	Child	-	-	-	0.026	0.043	0.126	0.037	-	-	-	-	0.021	-	-	0.029	0.038	0.035	0.046	0.042	0.043	-	0.020	0.034	0.025
Zn	Adult	0.014	0.005	0.004	-	0.004	0.012	0.003	-	0.002	0.003	-	-	0.002	-	0.004	-	0.003	0.004	0.008	0.007	-	0.003	-	0.008
	Child	0.021	0.007	0.006	-	0.005	0.018	0.005	-	0.003	0.004	-	-	0.003	-	0.006	-	0.005	0.006	0.013	0.011	-	0.004	-	0.012
Fe	Adult	0.010	0.005	0.009	0.008	0.009	0.055	-	0.012	0.010	0.031	0.032	0.041	0.006	0.009	0.018	-	0.005	0.011	0.011	0.030	0.020	0.045	-	0.017
	Child	0.015	0.007	0.014	0.012	0.013	0.084	-	0.019	0.015	0.047	0.049	0.062	0.009	0.014	0.027	-	0.007	0.017	0.017	0.045	0.031	0.068	-	0.025
NO_3^-	Adult	1.053	1.600	0.019	1.232	0.349	0.263	0.878	1.200	0.537	0.875	0.143	0.078	0.454	1.543	0.576	3.202	4.523	1.788	1.215	2.572	0.003	1.119	0.417	6.135
	Child	1.598	2.430	0.029	1.871	0.529	0.399	1.333	1.822	0.816	1.328	0.217	0.119	0.689	2.342	0.875	4.862	6.868	2.716	1.844	3.906	0.004	1.699	0.633	9.317

"–" not calculated

Table S3. The hazard quotient *via* dermal route (HQ_{dermal}) expressed as value x 10^{-3} for adults and children of trace elements and nitrate in selected rural Roma communities.

HQ	Person	P1	P2	P3	P4	P5	P6	P7	P8	P9	P10	P11	P12	P13	P14	P15	P16	P17	P18	P19	P20	P21	P22	P23	P24
Cr	Adult	-	8.00	-	-	-	-	-	8.13	4.13	-	-	7.33	-	-	-	-	-	5.47	8.13	3.07	-	-	31.47	5.87
	Child	-	4.67	-	-	-	-	-	4.80	2.40	-	-	4.27	-	-	-	-	-	3.20	4.80	1.73	-	-	18.40	3.47
Ni	Adult	0.19	0.73	-	0.11	0.09	0.15	0.10	0.10	-	0.08	-	0.11	-	-	0.10	0.19	0.10	0.20	0.11	0.40	-	0.08	-	0.24
	Child	0.11	0.43	-	0.06	0.05	0.09	0.06	0.06	-	0.05	-	0.08	-	-	0.05	0.11	0.06	0.11	0.06	0.24	-	0.05	-	0.14
As	Adult	-	-	-	-	0.98	1.79	2.42	-	-	-	-	-	1.33	1.61	-	1.54	1.61	-	1.47	1.93	3.61	3.02	2.56	4.32
	Child	-	-	-	-	0.60	1.05	1.44	-	-	-	-	-	0.81	0.95	-	0.91	0.95	-	0.88	1.12	2.14	1.75	1.51	2.53
Pb	Adult	-	0.07	-	-	-	-	-	-	-	-	-	-	-	-	-	-	-	-	0.05	-	-	-	-	0.05
	Child	-	0.05	-	-	-	-	-	-	-	-	-	-	-	-	-	-	-	-	0.02	-	-	-	-	0.02
Cd	Adult	-	-	-	-	-	-	1.20	-	-	-	-	-	-	0.80	-	2.40	0.80	0.80	-	-	-	-	-	0.80
	Child	-	-	-	-	-	-	0.80	-	-	-	-	-	-	0.40	-	1.20	0.40	0.40	-	-	-	-	-	0.40
Mn	Adult	-	78.74	0.40	7.04	2.13	41.25	2.45	7.42	0.96	1.81	1.17	2.74	1.81	1.56	1.17	-	-	1.48	0.78	10.17	98.45	78.49	1.48	28.60
	Child	-	46.25	0.23	4.14	1.25	24.23	1.44	4.35	0.56	1.06	0.69	1.61	1.06	0.92	0.69	-	-	0.86	0.46	5.97	57.83	46.10	0.88	16.80
Cu	Adult	-	-	-	0.18	0.30	0.88	0.26	-	-	-	-	0.14	-	-	0.20	0.27	0.24	0.32	0.29	0.30	-	0.14	0.24	0.17
	Child	-	-	-	0.11	0.18	0.52	0.15	-	-	-	-	0.08	-	-	0.12	0.16	0.14	0.19	0.17	0.18	-	0.08	0.14	0.10
Zn	Adult	0.22	-	0.06	-	0.06	0.18	0.05	-	0.04	-	-	-	0.03	-	0.06	-	0.05	0.06	0.13	0.11	-	0.04	-	0.13
	Child	0.13	0.05	0.04	-	0.03	0.11	0.03	-	0.02	0.03	-	-	0.02	-	0.04	-	0.03	0.03	0.08	0.07	-	0.02	-	0.08
Fe	Adult	0.34	0.17	0.33	0.28	0.30	1.96		0.44	0.36	1.09	1.14	1.44	0.21	0.32	0.63	-	0.16	0.38	0.39	1.04	0.72	1.59	-	0.59
	Child	0.20	0.10	0.19	0.16	0.18	1.15		0.26	0.21	0.64	0.67	0.84	0.13	0.19	0.37	-	0.09	0.23	0.23	0.61	0.42	0.93	-	0.35
NO_3^-	Adult	11.15	16.95	0.20	13.05	3.68	2.78	9.30	12.71	5.68	9.26	1.51	0.825	4.81	16.33	6.10	33.91	47.9	18.95	12.86	27.2	0.025	11.85	4.413	64.98
	Child	6.55	9.96	0.125	7.66	2.17	1.63	5.46	7.46	3.33	5.43	0.88	0.488	2.82	9.60	3.58	19.92	28.1	11.12	7.56	16.0	0.013	6.96	2.600	38.17

"–" not calculated

Table S4. The hazard index (HI) for adults and children of trace elements and nitrate in selected rural Roma communities

HI	Person	P1	P2	P3	P4	P5	P6	P7	P8	P9	P10	P11	P12	P13	P14	P15	P16	P17	P18	P19	P20	P21	P22	P23	P24
Cr	Adult	-	0.027	-	-	-	-	-	0.027	0.014	-	-	0.025	-	-	-	-	-	0.018	0.027	0.010	-	-	0.105	0.020
	Child	-	0.033	-	-	-	-	-	0.034	0.017	-	-	0.030	-	-	-	-	-	0.023	0.034	0.012	-	-	0.131	0.024
Ni	Adult	0.007	0.028	-	0.004	0.003	0.006	0.004	0.004	-	0.003	-	0.005	-	-	0.004	0.007	0.004	0.008	0.004	0.015	-	0.003	-	0.009
	Child	0.011	0.042	-	0.006	0.005	0.009	0.006	0.006	-	0.004	-	0.007	-	-	0.005	0.011	0.006	0.011	0.006	0.023	-	0.005	-	0.014
As	Adult	-	-	-	-	1.768	3.202	4.336	-	-	-	-	-	2.401	2.868	-	2.802	2.868	-	2.635	3.469	6.504	5.370	4.603	7.771
	Child	-	-	-	-	2.701	4.868	6.601	-	-	-	-	-	3.667	4.368	-	4.234	4.334	-	4.034	5.268	9.869	8.168	7.002	11.769
Pb	Adult	-	0.039	-	-	-	-	-	-	-	-	-	-	-	-	-	-	-	-	0.031	-	-	-	-	0.032
	Child	-	0.059	-	-	-	-	-	-	-	-	-	-	-	-	-	-	-	-	0.047	-	-	-	-	0.049
Cd	Adult	-	-	-	-	-		0.011		-	-	-	-	-	0.007	-	0.024	0.007	0.011	-	-	-	-	-	0.011
	Child	-	-	-	-	-		0.015		-	-	-	-	-	0.010	-	0.033	0.010	0.014	-	-	-	-	-	0.014
Mn	Adult	-	0.673	0.003	0.060	0.018	0.353	0.021	0.063	0.008	0.015	0.010	0.023	0.015	0.013	0.010	-	-	0.013	0.007	0.087	0.842	0.671	0.013	0.245
	Child	-	0.949	0.005	0.085	0.026	0.497	0.029	0.089	0.012	0.022	0.014	0.033	0.022	0.019	0.014	-	-	0.018	0.009	0.123	1.187	0.946	0.018	0.345
Cu	Adult	-	-		0.010	0.017	0.051	0.015	-	-	-	-	0.008	-	-	0.012	0.015	0.014	0.018	0.017	0.017	-	0.008	0.014	0.010
	Child	-	-		0.026	0.043	0.126	0.037	-	-	-	-	0.021	-	-	0.029	0.039	0.035	0.046	0.042	0.043	-	0.020	0.034	0.025
Zn	Adult	0.014	0.005	0.004	-	0.004	0.012	0.003	-	0.002	0.003	-	-	0.002	-	0.004	-	0.003	0.004	0.008	0.007	-	0.003	-	0.008
	Child	0.021	0.007	0.006	-	0.006	0.018	0.005	-	0.003	0.005	-	-	0.003	-	0.006	-	0.005	0.006	0.013	0.011	-	0.004	-	0.012
Fe	Adult	0.010	0.005	0.010	0.008	0.009	0.057	-	0.013	0.011	0.032	0.033	0.042	0.006	0.009	0.018	-	0.005	0.011	0.011	0.031	0.021	0.047	-	0.017
	Child	0.015	0.008	0.014	0.012	0.013	0.085	-	0.019	0.016	0.047	0.050	0.063	0.009	0.014	0.027	-	0.007	0.017	0.017	0.045	0.031	0.069	-	0.026
NO_3^-	Adult	1.064	1.617	0.020	1.245	0.352	0.266	0.887	1.213	0.543	0.884	0.145	0.079	0.459	1.559	0.582	3.236	4.571	1.807	1.228	2.599	0.003	1.131	0.422	6.200
	Child	1.605	2.440	0.030	1.879	0.532	0.401	1.338	1.829	0.819	1.334	0.218	0.119	0.692	2.352	0.879	4.882	6.896	2.727	1.852	3.922	0.004	1.706	0.636	9.355

“–“ not calculated

Table S5. The cancer risk (CR) for adults and children of As *via* ingestion and dermal routes in selected rural Roma communities

Person	CR *via* ingestion x 10^{-4}																							
	P1	P2	P3	P4	P5	P6	P7	P8	P9	P10	P11	P12	P13	P14	P15	P16	P17	P18	P19	P20	P21	P22	P23	P24
Adult	-	-	-	-	0.8	1.44	1.95	-	-	-	-	-	1.08	1.29	-	1.26	1.29	-	1.18	1.56	2.93	2.42	2.07	3.50
Child	-	-	-	-	1.22	2.19	2.97	-	-	-	-	-	1.65	1.97	-	1.91	1.95	-	1.82	2.37	4.44	3.68	3.15	5.30
	CR *via* dermal exposure x 10^{-6}																							
Adult	-	-	-	-	1.01	1.84	2.48	-	-	-	-	-	1.37	1.66	-	1.58	1.66	-	1.51	1.98	3.71	3.10	2.63	4.43
Child	-	-	-	-	0.61	1.01	1.48	-	-	-	-	-	0.83	0.97	-	0.94	0.97	-	0.90	1.15	2.20	1.80	1.55	2.59

"–" not calculated
